# Supplementary material for: Estimating associations between antidepressant use and incident mild cognitive impairment in older adults with depression
Source: PLoS One. 2020 Jan 17;15(1):e0227924. doi: 10.1371/journal.pone.0227924 (PMC6968868; doi:10.1371/journal.pone.0227924)
Supplement: S2 Table — (DOCX) [file pone.0227924.s002.docx]

**S2. The number and percentage of participants who met each of the criteria for depression in the first sensitivity analysis.**

| **Criterion** | **Total**  **(n=307)** |
| --- | --- |
| Self-reported active depression in the last two years | 292 (95.1%) |
| Clinical diagnosis of active depression based on current UDS examination and the clinician’s best judgement | 276 (89.9%) |
| Depression or dysphoria symptoms as reported by a coparticipant on the NPI-Q | 247 (80.5%) |
| GDS-15 score of at least 6 | 124 (40.4%) |
| Clinically depressed mood based on clinician interview | 94 (30.6%) |
